# Challenges in identifying indigenous peoples in population oral health surveys: a commentary

**DOI:** 10.1186/s12903-021-01455-w

**Published:** 2021-04-28

**Authors:** Lisa Jamieson, Joanne Hedges, Marco A. Peres, Carol C. Guarnizo-Herreño, João L. Bastos

**Affiliations:** 1 Australian Research Centre for Population Oral Health, University of Adelaide Dental School, Adelaide, SA 5005 Australia; 2 National Dental Research Institute Singapore, 5 Second Hospital Ave, Singapore, 168938 Singapore; 3 Departamento de Salud Colectiva, Facultad de Odontología, Universidad Nacional de Colombia, Bogotá, Colombia; 4 Post-Graduate Program in Public Health, Federal University of Santa Catarina, Florianópolis, Brazil

**Keywords:** Indigenous, Identification, Surveys

## Abstract

There are currently 370 million persons identifying as indigenous across 90 countries globally. Indigenous peoples generally face substantial exclusion/marginalization and poorer health status compared with non-indigenous majority populations; this includes poorer oral health status and reduced access to dental services. Population-level oral health surveys provide data to set priorities, inform policies, and monitor progress in dental disease experience/dental service utilisation over time. Rigorously and comprehensively measuring the oral health burden of indigenous populations is an ethical issue, though, given that survey instruments and sampling procedures are usually not sufficiently inclusive. This results in substantial underestimation or even biased estimation of dental disease rates and severity among indigenous peoples, making it difficult for policy makers to prioritise resources in this area. The methodological challenges identified include: (1) suboptimal identification of indigenous populations; (2) numerator-denominator bias and; (3) statistical analytic considerations. We suggest solutions that can be implemented to strengthen the visibility of indigenous peoples around the world in an oral health context. These include acknowledgment of the need to engage indigenous peoples with all data-related processes, encouraging the use of indigenous identifiers in national and regional data sets, and mitigating and/or carefully assessing biases inherent in population oral health methodologies for indigenous peoples.

## Background

Health policy, priority setting and resource distribution is dependent upon evidence from robust, comprehensive, and accurate population-level data. Although true for all populations, it is especially salient in obtaining health support and resources for socially disadvantaged populations, given their vulnerability in terms of political and economic influence. Indigenous populations are one such group. Indigenous peoples are both inheritors and practitioners of unique cultures and ways of relating to people and the environment. By definition, they ‘have a historical continuity with pre-invasion and pre-colonial societies that developed on their territories’ [1]. Despite having been subject to violence, exclusion and imposition of different worldviews for centuries, indigenous persons have retained social, cultural, economic and political characteristics that are distinct from those of the dominant societies of which they are part [2]. Indigenous peoples have sought recognition of their identities, ways of life, as well as their right to traditional lands, territories and natural resources for years. Yet, throughout history, their rights have systematically been denied or violated [3]. Contemporary indigenous peoples, who number 370 million and are spread over 90 countries worldwide, are among the most disadvantaged and vulnerable groups globally [4]. For example, one-third of the world’s 900 million extremely poor people residing in rural locations identify as indigenous [4]. The international community, including the United Nations and almost all countries with a history of colonisation, now recognise that special measures are required to promote and protect indigenous peoples’ rights to maintain their distinct cultures and ways of life [2]. Despite their unique cultural backgrounds, indigenous peoples from around the world are unified in their over-representation of poor health outcomes and lower life expectancy that have mainly arisen from a lack of protection of their rights [5].

Those identifying as indigenous typically face increased barriers to social resources in all aspects of life, compared with non-Indigenous persons [6]. Their access to, and utilisation of, healthcare is usually less, their health status is often poorer, and their health-related data are frequently incomplete, inaccurate or missing [7, 8]. The burden of oral conditions—e.g., dental caries, periodontal disease, oral cancer—at a global level is both substantial and increasing [9, 10], particularly in low- and middle-income countries. For indigenous populations, where data are available, dental diseases are a leading cause of morbidity and poor health-related quality of life [11–14]. In addition, indigenous peoples often experience oral health problems in different ways according to their worldviews, and may implement distinct self-care strategies to deal with such issues, as a function of their traditional knowledge and because of barriers (for example, racism) in accessing more ‘Westernised’ dental care [15, 16].

In recent decades, pressure has been placed on the United Nations to both recognise and bring attention to the rights and wellbeing of indigenous peoples as being distinct to other minority groups. In 2007, this culminated in the United Nation’s Declaration on the Rights of Indigenous Peoples [13, 14]. This has also occurred in many countries with a history of colonisation, with perhaps the most recent being Norway’s official recognition of the Sami people as its indigenous population in 1990 [15, 16]. Despite this, and the pressures from many advocacy groups calling for improved systems and data relating to indigenous peoples, few countries accurately and comprehensively measure indigenous population-level estimates for any health outcome, let alone oral health outcomes [17–19]. The recently established ‘International Group for Indigenous Health Measurement’, a network of government and non-government representatives from Australia, Canada, New Zealand, and the United States, has published guidelines on how to best measure mortality among indigenous peoples [20], but evidence is lacking about general morbidity and subjective measures of health at a global level.

There are empirical examples of how inaccurate collection of indigenous status impacts health-related funding and policies. Ventura Santos and colleagues demonstrated how estimates of the number of children born to indigenous women in Brazil are frequently under-estimated in the official Brazilian Census surveys, due to many Census officials not being fluent in indigenous languages and relying on non-indigenous respondents to obtain this information [21]. These non-indigenous respondents are not fully aware of the indigenous women’s reproductive histories and consequently provide undercounts of their children to the Census bureau. Accurately estimating counts of children born to indigenous women is, however, crucially important to estimate fertility rates and to inform public policy. So-called ‘standardised’ procedures that are not as inclusive as might appear may exclude indigenous peoples and almost certainly bias estimates collected on their behalf.

## Main text

In the interests of building on these efforts, and to provide guidance for the estimation of dental disease-related factors, it is timely to consider some critical methodological issues to be addressed when producing population-level oral health statistics for indigenous populations globally. These have been separated into three key areas for this commentary: (1) sub-optimal identification of indigenous populations; (2) numerator-denominator bias and; (3) statistical analytic considerations.

1. Sub-optimal identification of indigenous populations

At a global level, many governments/organisations/institutions tasked with conducting population oral health surveys do not collect data on indigenous status [22]. This violates the principles of the United Nations’ Declaration on the Rights of Indigenous peoples regarding the right of indigenous peoples to be accurately and appropriately counted in a culturally-respectful manner [2, 23]. In Canada, there are few population health data sources that routinely include indigenous status [17]. Because of distrust by some minority groups towards previous unethical research procedures, Norway does not routinely include ethnicity in population-level health surveys, but does include geographical location as a proxy indicator [23]. Some national oral health surveys in Latin America capture ethnicity, which then enables identification of indigenous peoples, for example, oral health surveys, or general health surveys with an oral health component, in Colombia, Panama, Chile, Brazil, and Mexico [24]. The issue is that the samples are likely not nationally representative of indigenous peoples from each of these respective countries let alone each of the multiple ethnic groups that are part of the ‘indigenous’ category. For example, oral health surveys in Brazil include four key age-groups and urban areas only [25]. Oral health surveys that do not appear to have included indigenous status include countries in Africa, Asia, Europe, and the Middle East, bearing in mind, however, that many surveys do include a measure of ethnicity which might enable further identification of indigenous peoples. National oral health surveys that do include indigenous status are those carried out in New Zealand, Australia, Canada, and Brazil [26–29]. Some smaller, convenience data are available for the United States and Norway [30, 31]. In some of these countries, however, those residing in indigenous-only locations (for example, reservations or communities) or in isolated communities have never been included.

It is important to acknowledge that many countries, especially low- and middle-income countries, have fewer resources by which to conduct population oral health surveys in the first place, often using the World Health Organisation’s pathfinder or other survey approaches that utilise a non-probability sampling methodology [32]. But the WHO guidelines on ethical issues in public health surveillance make it clear that countries have an obligation to develop appropriate, feasible and sustainable public health surveillance systems. Specifically, surveillance systems should have a clear purpose and a plan for data collection, analysis, use, and dissemination based on relevant public health priorities [33].

Population oral health surveys that do include indigenous status often do not sample in a way that yields representative estimates of indigenous populations. For example, for national oral health surveys in Australia, sampling procedures typically exclude remote and very remote locations, in which a disproportionately higher proportion of Indigenous Australians reside. The surveys typically rely on a participant having an operating phone and/or internet connection, but these are not always possible for many Indigenous Australians. The collection of clinical dental examination data relies on the participant having the wherewithal to locate and travel to a local dental public health clinic; but no assistance with transportation is provided. Follow-up questionnaires require a postal address, but many Indigenous Australians change their residential addresses frequently in a given year [34]. The questions/instruments/scales used in surveys are typically based on Western values and constructs, with minimal attempts to test the validity in an indigenous context. Frequently, self-report questionnaires are not translated into indigenous languages, thereby reducing the potential participation of those who only speak such languages. This problem is exacerbated by a limited number of census takers able to speak and understand indigenous languages. Finally, those organising appointments, conducting dental examinations and collecting chairside data are almost always non-indigenous, which makes many participants who identify as indigenous feel anxious, judged and devalued. For reasons relating to past experiences of discrimination and stigma, many opt not to identify as indigenous [35].

2. Numerator-denominator bias

There are historic and contemporary barriers to enumerating indigenous populations in almost all countries. Some of the complexities include issues relating to self-identity compared with identification through tribal affiliation. Across countries and time, self-recognition, community recognition and government recognition of indigenous identity have changed substantially, simultaneously with changes in the stigma and confidence of identifying as indigenous [36]. This impacts on the quality of indigenous status reporting data, with many countries in Europe electing to not identify particular ethnic minority or indigenous groups, despite this being the only way to assess the impacts of initiatives to reduce indigenous inequities in health outcomes [37].

In particular, numerator-denominator bias arises when the means of classifying indigenous status in the numerator data (population oral health surveys) differs from that implemented to estimate denominator data (typically Census, which is used in weighting to ensure estimates are representative at the population level). Unless data linkage is utilised, there will always be a degree of numerator-denominator bias in the estimation of indigenous status in population health surveys. In many instances, this difference can be substantial, leading to large discrepancies in actual prevalence of disease and what is captured [38].

The best approach to address this bias is to improve data collection, so that indigenous data for both the numerator and the denominator are reliable and consistent. For many population oral health surveys, weighting is used to generate population-level estimates (i.e., the denominator). Census data is typically used for weighting, but indigenous identification in Census data is not always accurate (for example, higher item non-response) [39]. Critically, a person’s indigenous status—which is almost always self-reported—is not always consistent across time, dependent in large part on the societal expectations and biases towards indigenous persons at a point in time [40, 41]. In terms of asking ethnic identity questions in population oral health surveys (the numerator), those collecting data need to be trained to use consistent questions (ideally matching those used in a government Census) and to deliver them in culturally sensitive ways, that is, in ways that are respectful of, and informed by, indigenous norms and expectations [42]. There are clear differences in self-reported indigenous status and indigenous status being attributed by the interviewer/examiner, with some historical health surveys conducted by non-indigenous persons who assumed a given participant’s indigenous status based on phenotypic characteristics, especially if such participants did not share the same first language as the examiner [43]. By definition, an indigenous person is one who belongs to an indigenous population through self-identification and who is recognized and accepted by that population as one of its members. This preserves for these communities the sovereign right to decide who belongs to them without external interference [8]. Indigenous status being attributed by an interviewer/examiner is a clear violation of these basic rights of what it means to be indigenous.

3. Statistical analytic considerations

Statistical analyses of population oral health data need to take into account inherent biases [44]. This includes, as an example, participation bias ~ consistent differences between those in a population who are invited to participate in a study and those who agree/refuse; this means the participating population differs from the whole population in a systematic way. In the case of indigenous data, care needs to be taken when using a comparator group, typically a broadly-structured ‘non-Indigenous’ group. Such analysis is necessary to highlight any inequities that thus provide evidence for improved delivery of health services and funding. However, it is important that a deficit model (framework that represents indigenous identity in a narrative of negativity, deficiency and failure [45]) is not always used and that an indigenous holistic view of health and wellbeing is included in any reporting, with examples including strengths-based approaches being used to counter both explicit and implicit deficit [46, 47]. It is also important to recognise the differences between indigenous groups within countries, for example, in the United States there are 574 culturally-diverse and federally-recognised tribes, with 229 in Alaska alone. These groups likely differ in important ways in their living conditions, culture and the capacity of governments to influence. Comparing the oral health of indigenous populations between countries is useful but often problematic because of: (1) marked historical, political, economic, social, and geographic differences, and; (2) differences in the quality and type of data collected, as well as survey methodologies used. Whilst important for global-level insight, care needs to be taken in the interpretation of any findings and how these results are then disseminated. For example, the ‘social determinants of health’ framework frequently used to analyse oral health inequalities typically focuses on social stratification by income, education and occupation. These groupings may be, in many cases, inappropriate for indigenous people, especially when making comparisons with non-indigenous groups because they do not take into account important historical, political and cultural impacts, for example, government policies of child removal, assimilation, removal of lands and rights, that exacerbate inequities over and above those described in the social determinants of health model [2].

Measuring inequality is of value when interpreting indigenous oral health data (either within or between countries), with care taken to include both absolute and relative measures because different conclusions can be drawn from the same data depending on the scale of measurement used [48]. It is especially beneficial when used to monitor trends over time or to determine the efficacy of a given intervention [49]. But there are inherent value judgements in the measurement of health inequalities, which are likely to differ markedly when considering indigenous compared with non-indigenous groups. For example, Harper and colleagues [50] describe how using a single measure of health inequality may implicitly endorse normative judgments, with such an endorsement being an unavoidable consequence of the structure of that given measure. This will have important ramifications for any policy initiatives that arise as a consequence of a given study’s findings. Population oral health surveys utilise weights so that population-level estimates can be generated, with the findings then considered to be ‘representative’. However, in almost all countries with indigenous populations, the age structure is younger than the non-indigenous population. To fully take this into account, standardisation should occur in addition to the weighting, preferably a population standard with an age distribution not dissimilar to the indigenous age distribution.

Time-trend analyses are important to measure oral health changes over time that have arisen due to policy changes, government initiatives or broader societal constructs (for example, social undesirability to have missing teeth). Time-trend analyses have revealed important information in, for example, declining rates of edentulism [51] and experiences of toothache [52]. But changes in the way indigenous status is captured in surveys used in time-series analyses leads to a bias in the outcomes. That is, supposed oral health trends over time among indigenous populations may be partially or wholly attributable to changes in indigenous identification as opposed to changes in oral health status/outcomes per se, as has been the case in linkage of hospital administrative data among indigenous populations [53].

The methodological issues discussed above may result in substantial under-estimation of indigenous dental disease rates, severity and incidence over time. This is not just a statistical issue, as such biases can lead to a lack of informed decision-making in terms of indigenous oral health initiatives, programs, and policies. One example is from the United States, where publicly-available national-level data from the National Health and Nutrition Examination surveys categorise race/ethnicity in terms of ‘Mexican–American’, ‘Other Hispanic’, ‘Non-Hispanic White’, ‘Non-Hispanic Black’, and ‘Others’; Alaskan Native/American Indian (AI/AN) is not included despite this group being the 6th largest minority (and the only indigenous group) in the country. This means population oral health estimates for AI/AN are not readily available, despite evidence from the Indian Health Service suggesting that the prevalence of untreated dental disease in this group is far higher than average US population estimates [54]. While much of NHANES data is used to inform health policy, particularly in regard to distribution of scarce health funding, the Indian Health Service in the US does have a range of operating funds for the provision of health care for Alaskan Natives and American Indians. Bearing in mind, however, that not all Alaskan Natives and American Indians choose to use the Indian Health Service [55]. Culturally-appropriate data governance structures, defined as being led and overseen by indigenous regulatory bodies, are increasingly being used in health research and health policy to improve the cultural safety and relevance of health initiatives for indigenous peoples [56, 57].

## Conclusions

The collection of indigenous status in oral health surveys varies enormously across countries; both in terms of the ways indigenous persons self-identify and the ways in which dental disease outcomes are assessed. At the national level of all countries in which indigenous populations reside, Census data, vital statistics data, and data from administrative health service data sets should include consistent indigenous identifiers. The participation/involvement of indigenous peoples in this harmonization process is key. There needs to be concerted efforts to improve the completeness and accuracy of indigenous data, especially in countries that may not have well-established data systems. While population data are often held at state or country level, culturally-appropriate data governance structures should be in place, with the use and sharing of any indigenous data overseen by a body of indigenous stakeholders.

## Data Availability

Not applicable.
